# Overarching Priorities for Health and Care Research in the United Kingdom: A Coproduced Synthesis of James Lind Alliance ‘Top 10s’

**DOI:** 10.1111/hex.14096

**Published:** 2024-06-19

**Authors:** Joanna C. Crocker, Lucy Moore, Margaret Ogden, Sally Crowe, Maaz Khan, Casper Schoemaker, Noémi B. A. Roy, Mark Taylor, Toto Gronlund, Teofila Bueser, Madeline Tatum, Benjamin Davies, Teresa Finlay

**Affiliations:** ^1^ Nuffield Department of Primary Care Health Sciences University of Oxford Oxford UK; ^2^ Lay Partner County Durham UK; ^3^ Crowe Associates Oxfordshire UK; ^4^ School of Clinical Medicine University of Cambridge Cambridge UK; ^5^ Oxford University Hospitals NHS Foundation Trust Oxford UK; ^6^ University Medical Center Utrecht Utrecht Netherlands; ^7^ National Institute for Health and Care Research (NIHR) Coordinating Centre Southampton UK; ^8^ James Lind Alliance Southampton UK; ^9^ South East Genomic Medicine Service Alliance Guy's & St Thomas' Hospital NHS Foundation Trust London UK; ^10^ Formerly Department for Continuing Education University of Oxford Oxford UK; ^11^ Department of Academic Neurosurgery University of Cambridge Cambridge UK

**Keywords:** carers, interdisciplinary research, patients, priority setting, research agenda, research funding, research priorities

## Abstract

**Introduction:**

James Lind Alliance (JLA) Priority Setting Partnerships (PSPs) produce ‘Top 10’ lists of health and care research priorities through a structured, shared decision‐making process with patients or service users, carers and health or care professionals who identify questions that are most important to them. To date, over 150 PSPs in different areas of health and care have published research priorities. Some PSPs share similar priorities, which could be combined, promoted and addressed through collaborative research to increase value and reduce research waste.

**Aim:**

The aim of this study was to identify overarching themes common to JLA PSP priorities across different areas of health and care.

**Methods:**

Our analysis included ‘Top 10’ research priorities produced by UK‐based JLA PSPs between 2016 and 2020. The priorities were coded deductively by the Health Research Classification System (HRCS) health category and research activity. We then carried out online workshops with patients, service users and carers to generate new codes not already captured by this framework. Within each code, multistakeholder inductive thematic analysis was used to identify overarching themes, defined as encompassing priorities from three or more PSPs covering two or more health categories. We used codesign methods to produce an interactive tool for end users to navigate the overarching themes.

**Results:**

Five hundred and fifteen research priorities from 51 PSPs were included in our analysis. The priorities together encompassed 20 of 21 HRCS health categories, the most common being ‘generic health relevance’ (22%), ‘mental health’ (18%) and ‘musculoskeletal’ (14%). We identified 89 overarching themes and subthemes, which we organised into a hierarchy with seven top‐level themes: quality of life, caregivers and families, causes and prevention, screening and diagnosis, treatment and management, services and systems and social influences and impacts.

**Conclusion:**

There are many overarching themes common to research priorities across multiple areas of health and care. To facilitate new research and research funding, we have developed an interactive tool to help researchers, funders and patients or service users to explore these priority topics. This is freely available to download online.

**Patient or Public Contribution:**

Patients or service users and carers were involved throughout the study, including deciding the aims, designing the study, analysing priorities to identify themes, interpreting and reporting the findings.

## 1 Background

Typically, researchers decide what research questions to answer, and funding organisations decide what research to fund. However, these decisions can differ markedly from the views of those grappling with health issues on the ground: people with lived experience (PLEx) of health conditions or service use—including patients and carers—and the professionals who diagnose and treat them [1, 2]. This mismatch can lead to reduced research value and increased research waste, as the end users of research are less likely to use or benefit from the findings [3]. In recent years, a variety of methods and models have been employed by some researchers and funders to include patients and other research users in setting research agendas [4, 5, 6, 7]. The James Lind Alliance (JLA) provides one of the most commonly published, structured approaches to patient involvement in research prioritisation [6, 8], aiming for research that is genuinely relevant and useful to end users.

The JLA is a UK–based, nonprofit initiative that brings together PLEx and health or care professionals in ‘Priority Setting Partnerships’ (PSPs) to identify which questions are most important for them to be answered by research [9]. PSPs usually focus on a particular health condition, specialty or care setting and are responsible for their own organisation and funding (which cannot be commercial), while the JLA provides guidance and facilitation. The National Institute for Health and Care Research (NIHR) funds the coordination of the JLA but does not usually fund JLA PSPs.

PSPs invite PLEx, health or care professionals and other stakeholders with relevant experience to submit questions they would like answering, usually via an online ‘harvesting survey’. These then go through a thorough process of review (including checking whether they are within the scope of the PSP and whether they have already been addressed by systematic reviews of existing evidence), grouping and interim prioritisation described in detail in the JLA Guidebook [9]. The process culminates with a consensus‐building workshop in which the top 20–30 prioritised questions are discussed and ranked by PLEx and health or care professionals to produce an agreed ‘Top 10’ list of research priorities.

Since the foundation of the JLA in 2004, over 150 PSPs have published ‘Top 10’ lists of research priorities, covering a wide range of health conditions and care specialties, and now include PSPs beyond the health and social care sector (e.g., adult social work and learning difficulties) [10]. The scope of PSPs has broadened, from focusing only on ‘treatment uncertainties’ (questions about treatment) in the earlier years of the JLA [11] to now identifying and prioritising ‘evidence uncertainties’ (not limited to treatment), the scope of which is determined by the PSP. Although most PSPs have been based in the United Kingdom, an increasing number of PSPs from other countries have published research priorities [11]. PSPs have reported a range of positive impacts of their work and ‘Top 10’ priorities, including a shift in research funding towards the issues that matter most to PLEx and health or care professionals, changes in organisational culture and benefits for the individuals involved [12, 13]. Challenges have also been reported for some PSPs, including time and financial resourcing, and issues related to inclusivity [14, 15, 16, 17].

With the large number of PSPs now in existence, there comes the opportunity to bring together the information they produce to answer broad questions about research prioritisation and the priorities themselves. PSPs naturally promote their ‘Top 10’ priorities to researchers and funders with a particular interest in their topic. However, some PSPs may share similar priorities, which could potentially be combined, promoted and addressed in collaborative ways. Likewise, researchers and funders with noncondition–specific remits may be interested in common priorities emerging across PSPs but may not have the resources to identify these themselves. The work presented in this paper aimed to identify overarching themes common to ‘Top 10’ research priorities from UK–based PSPs in different areas of health and care. It thus contributes to the JLA's wider aim of increasing the value of research to users and reducing research waste. It is part of a larger project undertaken to map and describe the nature of information published by PSPs and to describe the characteristics of ‘Top 10’ research priorities. The full project report is freely available via the lead author institution's website [18].

## 2 Methods

**Figure 1:**
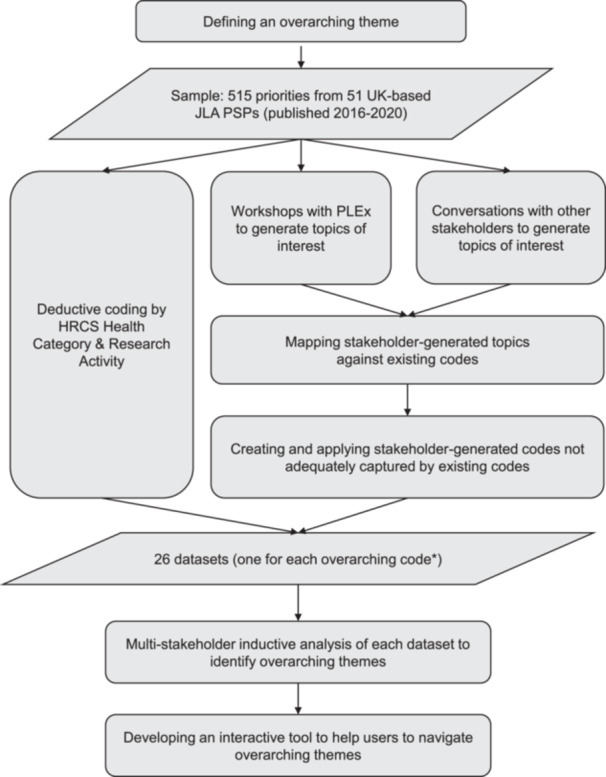
Overview of methods. *An overarching code was either an HRCS research activity or stakeholder‐generated code applied to priorities from at least three different PSPs from at least two different HRCS health categories (or ‘generic health relevance’).

Our methods are summarised in Figure 1.

### 2.1 Stakeholder Involvement

The project was a collaboration between people from a diverse range of stakeholder groups and perspectives within the United Kingdom and internationally. An advisory group was established by the project lead (J.C.C.) in Autumn 2020 and strongly influenced the direction, aims, design and findings of the project. The advisory group membership included:

- Four PLEx, all of whom had experience of involvement in PSPs and two of whom led a PSP (Juvenile Idiopathic Arthritis in the Netherlands and Spinal Cord Injury in Sweden);
- Three clinicians, two of whom had led a PSP (Degenerative Cervical Myelopathy and Rare Inherited Anaemias);
- Three patient and public involvement specialists, all of whom had experience supporting PSPs;
- Two health research funders with noncondition–specific remits;
- One academic health services researcher with a social science background.

They met three times during the project and also gave input via email correspondence and one‐to‐one or small group video calls. The PLEx and public involvement specialists were offered renumeration for their time (though not all chose to accept this).

The project was also informed by conversations with other potential end users including national research funders and research support staff. Later, 11 PLEx were involved in data analysis through online workshops as described below.

### 2.2 Defining an Overarching Theme

The objective of this study was to identify overarching themes common to ‘Top 10’ research priorities from UK–based PSPs in different areas of health and care. We (the project lead and advisory group) defined an ‘overarching theme’ as a theme or topic encompassing priorities from three or more PSPs and covering two or more health categories from the UK Clinical Research Collaboration (UKCRC) Health Research Classification System (HRCS). The HRCS was developed by the UKCRC Partners and was designed to produce a broad strategic overview of health research funding [19]. Since 2004, this framework has been extensively used by UK health research funders and internationally to classify and strategically assess award portfolios. The HRCS includes 21 separate health categories which encompass all diseases, conditions and areas of health. Each of the health categories includes research into both disease and normal function, and one health category—‘generic health relevance’—captures research that is relevant to all diseases and conditions or to general health and wellbeing. We therefore included ‘generic health relevance’ in our definition as a possible alternative to ‘two or more health categories’.

### 2.3 Sampling Priorities

We selected all ‘Top 10’ research priorities published by UK–based JLA PSPs between January 2016 and December 2020. This 5‐year cross section was obtained by working backwards chronologically in time from the end of 2020 (when this project began) to capture the most recent (and therefore likely most relevant) research priorities across a wide range of PSPs. We did not include PSPs that published priorities before 2016 due to limited time and resources. To facilitate coding and analysis, we assigned a unique ID number to each priority in our sample.

### 2.4 Deductive Coding by HRCS Health Category and Research Activity

We began by coding each priority by health category and research activity within the HRCS [19]. Health category was necessary as it formed part of our definition of an overarching theme. The HRCS also contains 48 research activity codes divided into eight broad code groups that encompass all aspects of health‐related research activity, ranging from basic to applied research (underpinning research; aetiology; prevention of disease and conditions; detection, screening and diagnosis; development of treatments and therapeutic interventions; evaluation of treatments and therapeutic interventions; management of diseases and conditions; health and social care services research). We agreed that coding priorities using this existing framework would be a helpful starting point for identifying overarching themes, particularly given its use among UK health research funders—a key group of end users of our project.

In addition to coding by HRCS health category and research activity, we also coded two other elements of priorities, where present: demographic subpopulation (age, gender, ethnicity or other) and outcome(s), using the Core Outcome Measures in Effectiveness Trials (COMET) taxonomy of outcomes [20]. This framework was designed to classify outcomes included in clinical trials; it contains 38 outcome domains divided into five core areas: death, physiological/clinical, life impact, resource use and adverse events.

Multiple codes from each framework could be assigned to a priority, where relevant. For example, the ‘Diabetes and Pregnancy’ PSP priorities were all coded under two HRCS health categories: ‘metabolic and endocrine’ and ‘reproductive health and childbirth’. In addition, one specific priority from this PSP which focussed on the ‘emotional and mental wellbeing needs of women with diabetes before, during and after pregnancy’ was also coded under ‘mental health’. Another priority that focussed on ‘the best way to test for and treat diabetes in late pregnancy’ was coded under two HRCS research activities: ‘evaluation of markers and technologies’ (Subcode 4.2) and ‘evaluation of treatments and therapeutic interventions’ (Code group 6).

Initially, each priority was coded by two members of the team (S.C.C. or M.K. and J.C.C.) independently. They used additional information concerning each priority, where available on the JLA website, to aid interpretation and coding decisions. They also used the HRCS online guidance [19] and developed their own tailored guidance specifically for JLA PSP priorities (File S1). These pairs of coders then met to compare codes, discuss the discrepancies and agree final codes. A third member of the team was consulted in the event of doubt or unresolved disagreement. Achieving consistently high levels of coding agreement without discussion remained challenging; so, after this initial period of intensive learning and quality improvement, a decision was made for three team members (S.C., M.K., L.M.) to continue independently double coding the entire sample of priorities. To reduce discussion time, a fourth member of the team (J.C.C.) acted as an adjudicator, comparing each pair of codes, and in the event of disagreement, whether to adopt one or both codes was decided by the fourth member. Coders were encouraged to attach explanatory comments to any code they had doubts about or felt were especially significant to support these decisions. Occasionally, the adjudicator proposed an entirely different code; this had to be discussed with at least one of the two original coders and could only be adopted if agreed. Each final code was therefore agreed upon by at least two of three coders.

### 2.5 Workshops With PLEx to Generate Topics of Interest

The HRCS framework has a predominantly clinical academic foundation and might not adequately capture the perspectives of PLEx. We therefore invited a group of 11 PLEx to take part in an online workshop in October 2021. These participants were suggested by members of the advisory group and the JLA Coordinating Team and were purposely diverse with regard to age, gender, ethnicity and geography within the United Kingdom. Almost all of them had experience with one or more JLA PSPs, either as a participant and/or steering group member—we deliberately sought this experience as we felt it was valuable given the focus of the project. Eight people were able to attend the workshop; the three people unable to attend had separate meetings with the project lead to ensure their views could be included. The workshop was professionally facilitated by a member of the advisory group with expertise in public involvement (S.C.), in collaboration with the project lead (J.C.C.,) and lay partner (M.O.). In preparation for the meeting, we asked the workshop participants to view a video presentation explaining the purpose of the project and the workshop, then review and familiarise themselves with 12 randomly selected research priorities from different PSPs within our sample. For each of these priorities, we provided a lay explanation and examples of original uncertainties from the PSPs (where available on the JLA website) and a glossary to explain any jargon. General information about PSPs and the JLA was also provided for context. During the workshop, we asked participants to work individually and in facilitated breakout groups to find pairs or groups of priorities that were similar in some way that was meaningful to them. We encouraged them to suggest labels or topics for these pairs and groups, as well as for any single priorities that stood out as important to them but could not be paired with another priority. With their permission, we audio recorded the discussions to enable all suggested topics to be accurately captured.

### 2.6 Conversations With Other Stakeholders to Generate Topics of Interest

In addition to the workshop with PLEx, the project lead also sought conversations with a variety of interested stakeholders to gather topic ideas. These included the project advisory group members, the JLA Coordinating Team and six national health research funders with a noncondition–specific remit. All but three funders responded to the email invitation and gave verbal input.

### 2.7 Mapping Stakeholder‐Generated Topics Against Existing Codes

We collated a total of 68 suggested topics from the workshop and conversations described above. We then mapped each suggested topic against the HRCS health category and research activity, COMET and subpopulation frameworks previously used to code priorities. The aim was to identify topics which were not adequately captured by these frameworks, either because they were absent from the frameworks or because they were divided across multiple categories within the frameworks (e.g., research about diet can be coded under any one of six different research activities in HRCS). This mapping process was carried out by the project lead (J.C.C.) with input from a lay partner (M.O.) and a member of the project advisory group with patient and public involvement expertise, experience facilitating JLA PSPs and a background in nursing (S.C.). They agreed to remove a small number of topics because they were not relevant to the main aim of the project or were too complex or difficult to assess (see File S2).

### 2.8 Creating and Applying Stakeholder‐Generated Codes

About half of the topics were thought to be adequately captured by the existing coding frameworks, while the other half were thought to merit the creation of a new code. Some of these new codes were combined because they were very similar. Table 1 lists all of the final new codes created. We refer to these as ‘stakeholder‐generated codes’ to distinguish them from the HRCS codes, COMET and subpopulation codes. The majority of these codes were contributed by PLEx from the workshops and advisory group. The project lead applied each new code to our sample of priorities in one of three ways: by keyword searching, by combining existing codes or by incorporating them into our inductive analysis of priorities, where they fell within an existing code. Details of code sources and application are provided in File S3.

**Table 1:** Stakeholder‐generated codes.

Alcohol
Caregivers & Families
Communication & information sharing
Delay
Diet
Exercise
Fatigue
Health education
Health inequalities
Health literacy
Inequality of access (to services)
Multi‐morbidity
Pain
Pharmaceuticals
Physical environment
Place of care
Psychological impact of illness
Psychological risk factors
Psychological, social, behavioural & economic determinants of health
Public knowledge, views, attitudes & behaviour
Quality of life
Research (design, methods, dissemination & implementation)
Resources and infrastructure
Self‐management (of conditions)
Sex & sexual health
Shared decision‐making
Smoking
Social (including any social angle on health/care)
Substance misuse
Surgery
Technology
Utilising patient/carer expertise

### 2.9 Multistakeholder, Inductive Analysis

The previous coding using existing frameworks and stakeholder‐generated codes resulted in a large number of data sets for analysis, each data set containing a list of research priorities assigned a certain code and spanning at least three PSPs from at least two HRCS health categories (or ‘generic health relevance’). In part due to limited resources, we made the practical decision to exclude the COMET and subpopulation data sets from analysis (with the exception of the COMET ‘Life Impact’ core area); as a large number of priorities did not fit into these two frameworks, many priorities lacked a specified outcome and most did not focus on a demographic subpopulation of PLEx. Additionally, where an outcome *was* specified, there was substantial overlap between the COMET and HRCS frameworks. By contrast, almost all research priorities had been successfully coded by HRCS research activity. We therefore included only the 26 data sets corresponding to HRCS research activity codes, the stakeholder‐generated codes and COMET Life Impact. We retained the latter because the workshop with PLEx highlighted the importance of research on quality of life, but this did not have its own distinct code within the HRCS research activity framework. Since multiple coding had been applied, the same research priority could appear in more than one data set.

To identify overarching themes, the project lead (J.C.C.) carried out an inductive thematic analysis on each of these data sets, informed by the ‘one sheet of paper’ method [21]. For each data set, possible themes were brainstormed on a blank sheet of paper, along with ID numbers of the relevant priorities, which were visibly linked together when originating from the same PSP. Similar/related themes were clustered together and combined where appropriate to meet the criterion of covering three or more PSPs. For themes meeting this criterion, the underlying priorities were reviewed again to determine whether they met the second criterion of covering two or more HRCS health categories or ‘generic health relevance’. A second team member (‘second reviewer’) also reviewed each data set independently of the project lead and proposed their own overarching themes. The second reviewers comprised six members of the project advisory group (C.S., M.K., M.Tay., N.B.A.R., T.B., T.G.) and one postgraduate student with a background in public health and expertise in translational health sciences (M.Tat.). Following comparison of the two sets of overarching themes, the project lead proposed a revised set of overarching themes; these were presented to the second reviewers and wider project advisory group for feedback, and further revisions were made until arriving at an agreed set of overarching themes.

Part‐way through the inductive analysis process, the project advisory group met and worked in small groups to discuss what makes a ‘good’ or ‘bad’ overarching theme (from a user perspective), whether information (such as frequency across PSPs, ranking of priorities) matters and what else might be important in interpreting the findings. Overarching themes proposed by the project lead and second reviewers were anonymised and used as examples, and the second reviewers did not work on their own suggested themes. This allowed for open discussion and a degree of impartiality in the small group work. The discussions informed the remaining analysis, development and presentation of overarching themes.

### 2.10 Developing an Interactive Tool

We determined that the primary end users of our overarching themes were researchers (including service user researchers) and research funders with an interest in health and care. We also wanted to ensure they were accessible to PLEx and other lay users. To help these different users navigate the large number of overarching themes, we worked with Design Science (a communication design agency specialising in science, education and health based in London, United Kingdom) to develop a user‐friendly and interactive PDF tool. First, we aimed to group the overarching themes into fewer than 10 top‐level themes, which would serve as meaningful and intuitive entry points to the overarching themes and allow users to focus on the areas of particular interest to them. This grouping was an iterative process involving the advisory group and two further PLEx workshops in the summer of 2022. During these workshops, we showed participants of the previous PLEx workshop how their work had influenced our analysis and findings and sought their feedback on the draft top‐level themes and our plan for the tool. The draft tool was also reviewed by nine members of the advisory group and six other potential users including health and social science researchers, members of NIHR Research Design Service and a public involvement professional. They provided general and page‐specific feedback via a structured form. Based on this feedback, several aspects of the tool were revised including content, layout, language and graphics.

## 3 Results

### 3.1 PSPs and HRCS Health Categories

**Figure 2:**
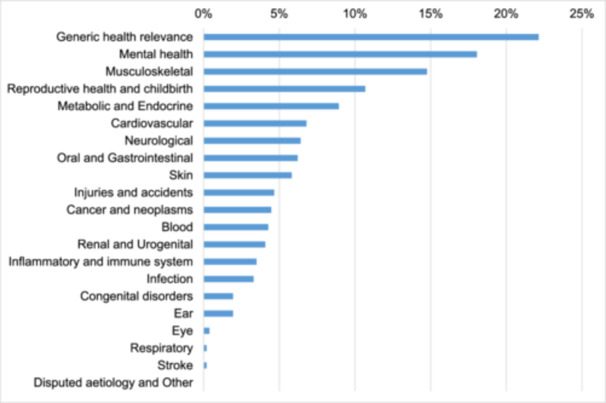
Research priorities by HRCS health category (*N* = 515).

Our sample consisted of 515 priorities from 51 UK–based JLA PSPs, listed in Table 2. The priorities were distributed across 20 of the 21 HRCS health categories as shown in Figure 2 (the only health category not covered was ‘Disputed Aetiology and Other’, which is rarely used according to HRCS). The most common health category among JLA priorities was ‘generic health relevance’ (*N* = 114; 22%), which includes research applicable to all diseases and conditions or to the general health and wellbeing of individuals; its high frequency is largely due to the 10 PSPs in our sample focussed on noncondition–specific services or populations. The high frequency of ‘mental health’ (18.1%) is due to a combination of 60 priorities from six PSPs focussed on mental health conditions and 32 priorities (from 21 other PSPs) relating to the psychological wellbeing or subgroups of people with physical health conditions. ‘Musculoskeletal’ was the third largest category (14.8%), comprising 75 priorities from seven PSPs focussed on musculoskeletal issues and only one priority from another PSP (Bleeding Disorders).

**Table 2:** UK–based JLA PSPs included in our sample.

Adult Social Work (England only)	Heart Surgery
Advanced Heart Failure	Hyperacusis
Alcohol‐related liver disease	Hyperhidrosis
Autism	Idiopathic Intracranial Hypertension
Bipolar	Kidney Transplant
Bleeding Disorders	Learning Difficulties (Scotland only)
Blood Pressure in Pregnancy	Lichen Sclerosus
Blood Transfusion and Blood Donation	Living With and Beyond Cancer
Broken Bones in older people	Mental Health in Children and Young People
Broken Bones of the Upper Limb in People over 50 (Fractures of the Shoulder, Arm or Wrist)	Miscarriage
Cellulitis	Mitochondrial Disease
Coeliac Disease	Multiple Conditions in Later Life
Common Conditions Affecting the Hand & Wrist	Nutritional Screening and Malnutrition
Contraception	Occupational Therapy
Cystic Fibrosis	Oral and Dental Health
Degenerative Cervical Myelopathy	Paediatric Lower Limb Surgery
Depression	Patient Safety in Primary Care
Detecting Cancer Early	Pessary use for Prolapse
Diabetes & Pregnancy	Physiotherapy
Diabetes (Type 2)	Psoriasis
Digital Technology for Mental Health	Rare Inherited Anaemias
Early Hip & Knee Osteoarthritis	Rare Musculoskeletal Disease in Adulthood
Electronic Cigarettes	Revision Knee Replacement
Emergency Medicine	Safe Care for Adults with Complex Health Needs
Endometriosis	Scoliosis
Foot Health	

### 3.2 Overarching Themes

**Figure 3:**
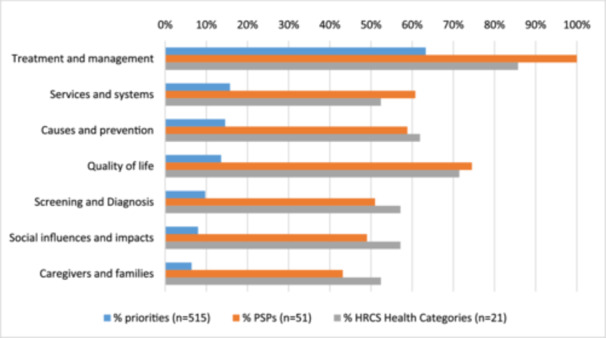
Frequency of top‐level overarching themes.

We identified a total of 89 overarching themes and subthemes, arranged in a taxonomy with seven top‐level themes: treatment and management,; services and systems, causes and prevention, quality of life, screening and diagnosis, social influences and impacts and caregivers and families (see Table 3 and Figure 3). All 89 overarching themes are listed in Table 4 along with their frequency in our sample of priorities. Underlying data for each overarching theme can be accessed via our interactive tool [22].

**Table 3:** Description of top‐level themes, with examples.

Top‐level theme	Description	Example priorities (with PSP in brackets)
Quality of life	Research to understand, improve or maintain people's general wellbeing or the degree to which they are healthy, comfortable and able to enjoy the activities of daily living. This includes psychological and emotional wellbeing, social and economic wellbeing and physical functioning.	How is quality of life affected by rare anaemia and its treatment? How could this be improved for patients? (Rare Inherited Anaemias) How are the psychological impacts (including on body image) of diagnosis and treatment best managed? (Scoliosis) What are the best interventions to keep people with early osteoarthritis working? (Early Hip & Knee Osteoarthritis)
Caregivers and families	Research concerning the caregivers and families of people with lived experience of a health condition. We define caregivers as people who regularly look after someone, whether formal/paid workers or informal/unpaid family members or volunteers.	What are the best ways to help friends and family members to support people with depression? (Depression) In what ways can carers of older people with multiple conditions be supported to maintain their own physical and psychological wellbeing? (Multiple Conditions in Later Life) How can (paid and unpaid) carers' knowledge of a person with complex health needs and their specific healthcare needs be recognised and used to improve and inform the care provided by professionals? (Safe Care for Adults with Complex Health Needs)
Causes and prevention	Research to understand the causes of health conditions and health behaviour, and how to prevent them from occurring or reoccurring. These include (but are not limited to) biological, psychological, social, environmental and lifestyle factors.	What is the cause of pregnancy hypertension (including pre‐eclampsia)? (Blood Pressure in Pregnancy) What can be done to prevent rare metabolic bone disorders in the first place, or to stop them from getting worse? (Rare Musculoskeletal Disease in Adulthood) How can we better understand the associations between coeliac disease and other conditions, for example, Type 1 diabetes and autoimmune thyroid disease, and what factors influence the risk of developing such conditions? (Coeliac Disease)
Screening and diagnosis	Research concerning the screening for, and diagnosis of, conditions. This includes patients' experiences of screening and diagnosis, and the development and evaluation of tests, tools and techniques.	Why does it take so long to get a diagnosis of bipolar disorder, and how could the time to diagnosis be shortened? (Bipolar) What are the early signs and symptoms of cellulitis that can help to ensure speedy treatment? (Cellulitis) How important are specialised tests (such as diagnostic ultrasound imaging/advanced vascular and gait/functional assessment) learned at the postgraduate level, in the diagnosis of foot health problems? (Foot Health)
Treatment and management	Research concerning the treatment or management of conditions, including identifying, evaluating and optimising therapeutic interventions and management strategies.	What are the best strategies to optimise communication of information between patients/carers and clinicians to enable shared decision‐making? (Paediatric Lower Limb Surgery) Can novel therapies, including stem‐cell, gene, pharmacological and neuroprotective therapies, be identified to improve the health and wellbeing of people living with DCM and slow down disease progression? (Degenerative Cervical Myelopathy) How can immunosuppression be personalised to individual patients to improve the results of transplantation? (Kidney Transplant)
Services and systems	Research to understand and improve the design, delivery and functioning of health and care services and systems.	Does partnership working between adult social workers and other health and social care professionals result in better outcomes for people using services? (Adult Social Work) How can occupational therapy services be more inclusive of both mental and physical health? (Occupational Therapy) What is the most effective way of training teachers and other staff in schools and colleges to detect early signs of mental health difficulties in children and young people? (Mental Health in Children and Young People)
Social influences and impacts	Research to understand and address the social and societal influences on conditions and behaviour, and the social impacts of living with conditions.	What would encourage more people (especially black and ethnic minority groups or people with a rare blood type) to donate blood? (Blood Transfusion and Blood Donation) How can we encourage employers to apply person‐centred interventions and support to help autistic people maximise their potential and performance in the workplace? (Autism) Does the stigma associated with alcohol misuse affect the willingness of people with alcohol‐related liver disease to ask for help? (Alcohol‐Related Liver Disease)

**Table 4:** Frequencies of overarching themes.

Overarching theme	No. of priorities	% Priorities (*N* = 515)	No. of PSPs	% PSPs (*N* = 51)	No. of health categories^a^	% Health categories^a^ (*N* = 21)
**1 Quality of life**	**70**	**13.6**	**38**	**74.5**	**15**	**71.4**
1.1 General quality of life	23	4.5	18	35.3	12	57.1
1.1.1 Understanding the impact of conditions on general quality of life	9	1.7	9	17.6	8	38.1
1.1.2 Improving and maintaining general quality of life	16	3.1	12	23.5	9	42.9
1.2 Psychological and emotional wellbeing	28	5.4	21	41.2	10	47.6
1.2.1 Understanding psychological & emotional wellbeing	15	2.9	15	29.4	9	42.9
1.2.1.1 Understanding psychological & emotional impacts of conditions	9	1.7	9	17.6	7	33.3
1.2.1.2 Understanding psychological and emotional impacts of treatments	5	1.0	5	9.8	4	19.0
1.2.2 Improving and maintaining psychological and emotional wellbeing	22	4.3	16	31.4	8	38.1
1.3 Social and economic wellbeing	16	3.1	12	23.5	9	42.9
1.3.1 Understanding social and economic impacts of conditions (also 7.2)	6	1.2	6	11.8	6	28.6
1.3.2 Supporting participation and integration in society (also 7.3)	11	2.1	8	15.7	7	33.3
1.3.2.1 Supporting work and employment (also 7.3.1)	3	0.6	3	5.9	2	9.5
1.4 Physical functioning	13	2.5	7	13.7	6	28.6
1.4.1 Understanding the impacts of conditions on physical functioning	3	0.6	3	5.9	3	14.3
1.4.2 Reducing the impacts of conditions on physical functioning	10	1.9	5	9.8	4	19.0
**2 Caregivers and families**	**33**	**6.4**	**22**	**43.1**	**11**	**52.4**
2.1 Empowering caregivers and families	7	1.4	6	11.8	3	14.3
2.2 Understanding the influence of family (also 7.1.1 and 3.1.3.1)	3	0.6	3	5.9	2	9.5
2.3 Understanding the impact on caregivers and families	7	1.4	6	11.8	6	28.6
2.4 Supporting the wellbeing of caregivers and families	9	1.7	9	17.6	8	38.1
2.5 Improving professional engagement and communication with caregivers and families	12	2.3	9	17.6	7	33.3
**3 Causes and prevention**	**75**	**14.6**	**30**	**58.8**	**13**	**61.9**
3.1 Understanding the causes of health conditions and health behaviour	45	8.7	22	43.1	12	57.1
3.1.1 Understanding biological mechanisms and influences on health	18	3.5	11	21.6	10	47.6
3.1.1.1 Understanding genetic influences	6	1.2	4	7.8	4	19.0
3.1.1.2 Understanding hormonal influences	3	0.6	3	5.9	3	14.3
3.1.2 Understanding psychological influences on health	4	0.8	4	7.8	4	19.0
3.1.3 Understanding social influences on health and health behaviour (also 7.1)	9	1.7	9	17.6	5	23.8
3.1.3.1 Understanding the influence of family (also 2.2 and 7.1.1)	3	0.6	3	5.9	2	9.5
3.1.4 Understanding environmental and lifestyle influences on health	5	1.0	5	9.8	4	19.0
3.1.5 Understanding how and why a condition progresses	11	2.1	8	15.7	7	33.3
3.1.6 Understanding how conditions affect various population groups differently	6	1.2	6	11.8	6	28.6
3.2 Preventing health conditions from (re)occurring	29	5.6	16	31.4	8	38.1
3.2.1 Modifying lifestyle for prevention	8	1.6	4	7.8	4	19.0
3.3 Understanding and preventing multi‐morbidity	9	1.7	7	13.7	6	28.6
**4 Screening and diagnosis**	**50**	**9.7**	**26**	**51.0**	**12**	**57.1**
4.1 Reducing time to diagnosis	20	3.9	14	27.5	10	47.6
4.2 Finding the best test, tool or method for screening and diagnosis	35	6.8	22	43.1	12	57.1
4.2.1 Noninvasive techniques (unspecified)	3	0.6	3	5.9	4	19.0
4.2.2 Imaging techniques	6	1.2	5	9.8	4	19.0
4.2.3 Blood tests	5	1.0	5	9.8	5	23.8
**5 Treatment and management**	**326**	**63.3**	**51**	**100.0**	**18**	**85.7**
5.1 Improving communication and information sharing between people with lived experience and professionals	20	3.9	16	31.4	9	42.9
5.1.1 Enabling and improving shared decision‐making	8	1.6	8	15.7	6	28.6
5.2 Identifying and evaluating treatments and therapeutic interventions	228	44.3	49	96.1	17	81.0
5.2.1 Identifying and developing new treatments	27	5.2	17	33.3	12	57.1
5.2.1.1 Identifying and developing pharmaceutical treatments	7	1.4	6	11.8	6	28.6
5.2.1.2 Identifying and developing cellular and gene therapies	7	1.4	5	9.8	5	23.8
5.2.1.3 Finding a cure	4	0.8	4	7.8	5	23.8
5.2.2 Evaluating treatments and therapeutic interventions	210	40.8	48	94.1	16	76.2
5.2.2.1 Finding the best treatment, therapy or management strategy	76	14.8	37	72.5	16	76.2
5.2.2.2 Evaluating the cost effectiveness of treatments and interventions	19	3.7	9	17.6	4	19.0
5.2.2.3 Evaluating the benefits and risks of pharmaceuticals	25	4.9	18	35.3	12	57.1
5.2.2.4 Evaluating the benefits and risks of cellular and gene therapies	3	0.6	3	5.9	3	14.3
5.2.2.5 Evaluating the benefits and risks of medical devices	17	3.3	6	11.8	6	28.6
5.2.2.6 Evaluating the benefits and risks of surgical interventions	39	7.6	16	31.4	8	38.1
5.2.2.7 Evaluating the benefits and risks of psychological and behavioural interventions	21	4.1	15	29.4	10	47.6
5.2.2.8 Evaluating the benefits and risks of physical interventions	33	6.4	18	35.3	9	42.9
5.2.2.9 Evaluating the benefits and risks of digital technologies	14	2.7	8	15.7	8	38.1
5.2.2.10 Improving how interventions are evaluated	9	1.7	9	17.6	4	19.0
5.3 Monitoring, predicting and preventing disease	40	7.8	27	52.9	14	66.7
5.3.1 Monitoring and assessing disease	18	3.5	15	29.4	11	52.4
5.3.2 Preventing deterioration and complications	19	3.7	17	33.3	11	52.4
5.3.3 Predicting deterioration, complications and treatment response	10	1.9	9	17.6	6	28.6
5.4 Improving self‐management of conditions	51	9.9	29	56.9	16	76.2
5.4.1 Modifying lifestyle for self‐management	24	4.7	19	37.3	14	66.7
5.4.1.1 Using diet to manage health	11	2.1	10	19.6	10	47.6
5.4.1.2 Using exercise to manage health	15	2.9	13	25.5	10	47.6
5.5 Tailoring care to individuals or subgroups	36	7.0	23	45.1	13	61.9
5.5.1 Managing frailty	5	1.0	4	7.8	2	9.5
5.6 Managing symptoms and side‐effects	21	4.1	11	21.6	9	42.9
5.6.1 Managing pain	7	1.4	6	11.8	6	28.6
5.6.2 Managing fatigue	6	1.2	6	11.8	6	28.6
5.6.3 Managing side‐effects of treatment	9	1.7	5	9.8	5	23.8
5.7 Improving rehabilitation following injury or surgery	7	1.4	6	11.8	4	19.0
5.8 Considering or avoiding surgery	12	2.3	10	19.6	6	28.6
5.9 Managing multimorbidity	34	6.6	13	25.5	9	42.9
5.9.1 Managing physical and mental comorbidity	5	1.0	5	9.8	3	14.3
**6 Services and systems**	**81**	**15.7**	**31**	**60.8**	**11**	**52.4**
6.1 Optimising multiagency and multiprofessional coordination	20	3.9	14	27.5	6	28.6
6.2 Ensuring safety	13	2.5	5	9.8	3	14.3
6.3 Improving access to services	10	1.9	7	13.7	5	23.8
6.4 Achieving holistic/person‐centred care	9	1.7	8	15.7	5	23.8
6.4.1 Improving palliative and end‐of‐life care	3	0.6	3	5.9	3	14.3
6.5 Understanding and reducing delays/waiting times	4	0.8	4	7.8	3	14.3
6.6 Improving how health information is recorded	3	0.6	3	5.9	2	9.5
6.7 Training and developing professionals	6	1.2	5	9.8	5	23.8
**7 Social influences and impacts**	**41**	**8.0**	**25**	**49.0**	**12**	**57.1**
7.1 Understanding social influences on health and health behaviour (also 3.1.3)	9	1.7	9	17.6	5	23.8
7.1.1 Understanding the influence of family (also 2.2 and 3.1.3.1)	3	0.6	3	5.9	2	9.5
7.2 Understanding the social and economic impacts of conditions (also 1.3.1)	6	1.2	6	11.8	6	28.6
7.3 Supporting participation and integration in society (also 1.3.2)	11	2.1	8	15.7	6	28.6
7.3.1 Supporting work and employment (also 1.3.2.1)	3	0.6	3	5.9	2	9.5
7.4 Addressing health inequalities	8	1.6	7	13.7	4	19.0
7.5 Investigating public health awareness and attitudes	10	1.9	8	15.7	5	23.8

Abbreviation: PSPs, Priority Setting Partnerships.

^a^ Health categories from the UKCRC Health Research Classification System. There are 21 in total.

The seven top‐level themes are not mutually exclusive but can be considered different ‘windows’ into the overarching themes and PSP priorities or alternative ways of grouping them according to different perspectives. They sometimes overlap, with six overarching themes appearing under more than one of the seven top‐level themes. For example, ‘understanding the influence of family’ appears under three major themes: ‘causes and prevention’, ‘caregivers and families’ and ‘social influences and impacts’. Such overlap stems from allowing multiple coding of single priorities and avoiding prioritising certain stakeholder perspectives over others. Our findings are therefore relevant to a wide variety of users.

### 3.3 Interactive Tool

The overarching themes and their underpinning data can be explored using our interactive PDF tool, which includes specific examples of how the tool might be used by researchers, funders and PLEx. It was published online in April 2023 and is freely available to download online [22].

## 4 Discussion

### 4.1 Summary of Main Findings

In this study, we identified 89 overarching themes from UK PSP priorities published between 2016 and 2020. These ranged from basic science research into the causes of conditions to applied research concerning clinical practice and health systems. A downloadable interactive tool [22] enables users to navigate the overarching themes according to areas of interest.

Almost half of our overarching themes related to the treatment and/or management of conditions, which was also the focus of a majority of priorities in our sample. This likely reflects the historical focus of the JLA on ‘treatment uncertainties’; only in November 2018 did the JLA Guidebook change the terminology to ‘evidence uncertainties’ to ‘reflect the broader scope of many PSPs that include uncertainties around interventions that are beyond ‘treatments’, such as care, support and diagnosis' [9]. It may also reflect the applied health interests of those involved in PSPs (PLEx and health or care professionals). Although we sought to identify themes spanning multiple HRCS health categories, the ‘mental health’ category appeared in a large number of priorities from PSPs focussed on physical health conditions and could therefore be considered an overarching theme in its own right. Indeed, there is substantial overlap between this health category and our overarching theme ‘psychological and emotional wellbeing’, which includes priorities concerned with understanding the psychological and emotional impacts of physical conditions and treatments or improving and maintaining the psychological and emotional wellbeing of PLEx.

We demonstrated that the HRCS health categories and research activities can be successfully applied to priorities but also discovered the limitations of the HRCS with regard to themes of importance to PLEx and other nonacademic stakeholders. Involving these groups in the analysis was necessary to highlight certain topics which could otherwise have been missed, including health inequalities, shared decision‐making, self‐management, multimorbidity, holistic/person‐centred care and information sharing. Of particular note, our top‐level themes ‘quality of life’, ‘caregivers and families’ and ‘social influences and impact’ emerged as a direct result of stakeholder involvement.

In 2020, as this study was beginning, Levelink et al. published the findings of a systematic review of international academic literature reporting the identification and prioritisation of research priorities with substantial patient involvement [8]. The review included 34 papers and identified overarching themes across the reported research priorities, which were subsumed under nine main themes and 24 subthemes. Despite the broader scope of their review, the smaller number of included priorities and the little overlap with our sample (only four JLA PSPs were included in both studies), some similar main themes emerged, including ‘treatment’, ‘health care system’, ‘prevention’, ‘diagnosis’ and ‘(informal) carers’ [8]. This suggests that many of the overarching themes we identified may have persisted for some time and may be shared by PLEx and health or care professionals internationally. However, we also identified additional themes not shared by Levelink and colleagues' study, including our top‐level themes ‘quality of life’ and ‘social influences and impacts’ and many of our subthemes. This may be due to the larger number of priorities included, the UK focus and the coproduction of our work with PLEx and other nonacademic stakeholders (by contrast, only academic researchers were involved in Levelink and colleagues' systematic review).

### 4.2 Strengths and Limitations

Strengths of this study include the quantity and breadth of the PSPs and research priorities included in our analyses (spanning almost all HRCS health categories) and the rigorous process of coding and identification of overarching themes by a multidisciplinary team. We also involved a diverse group of PLEx in the analysis of research priorities; this provided rich new insights and ensured that several important topics were included in our overarching themes.

Our analysis focussed on a cross section of PSPs that published their priorities within a 5‐year window. It does not include more recent or older PSPs, which may have provided rich opportunities for the identification of further overarching themes. In particular, any significant shift in priorities since the COVID‐19 pandemic (e.g., related to changes in how healthcare is delivered and experienced) would not have been captured. We also did not seek to identify overarching themes relating to specific age, gender or ethnic groups, although coding by demographic subpopulation is available should others wish to explore this.

To aid accurate coding of research priorities, we referred to extra information about the priority (explanatory note, examples of original uncertainties and/or project report) where available on the JLA website. However, for many PSPs, this information was not available, leaving coders to rely on their own understanding of the priority. It is therefore possible that some of the research priorities were not assigned the most appropriate code. We hope to have reduced this likelihood by having two coders independently coding each priority. The identification of overarching themes was a highly creative process likely influenced by the analysts' backgrounds, experiences and perspectives. Again, the involvement of two team members in the inductive analysis of each data set will have reduced the influence of any single person and the likelihood of important themes being overlooked.

Finally, our overarching themes are only as robust as the ‘Top 10’ research priorities underpinning them. During our PLEx workshops, some participants highlighted the relative lack of research priorities addressing issues of particular importance to underrepresented or nontraditionally engaged communities. It was suggested that a broader range of themes might have been identified if PSP participants included more people from communities not usually engaged in research. The JLA acknowledges this and encourages new PSPs to plan how they will involve diverse and underserved populations meaningfully throughout the JLA process.

### 4.3 Implications for Researchers and Funders

We encourage researchers, funders and public contributors with a broad interest in health and care to explore the overarching themes using our interactive tool [22]. These themes could be used to inform decisions about which topics to target for research investment, providing a mechanism for understanding and incorporating the overarching priorities of PLEx and frontline professionals across a wide range of specialties. They could also help researchers and funders working in specific areas of health or care to identify other disciplines or specialties with similar priorities, thus fostering interdisciplinary collaboration and greater research efficiency. For example, the overarching theme ‘addressing health inequalities’ brings together research priorities in the very different areas of blood transfusion, contraception, mental health, nutrition, patient safety and physiotherapy.

Many priorities in our sample have not yet been individually addressed by the research community [23], but our findings provide a new opportunity to reconsider some of these priorities collectively. Health research is often conducted in siloes focusing on specific conditions or professions; we hope that our findings will inspire people to work together across these siloes to systemically address common, holistic issues of importance to people providing and receiving care. In addition, the prominence of priorities concerning mental health, which appeared across a significant number of PSPs focussed on physical health conditions, suggests a need for greater prioritisation and integration of mental health considerations across the health research landscape.

When using the overarching themes to inform decision‐making, we recommend that users refer to supporting information for the relevant PSPs published on the JLA website, including the explanatory note and original submissions underpinning each priority. This will help ensure that the priorities and themes are understood and interpreted correctly. We also advise working with PLEx and frontline health and care professionals so that their priorities continue to be reflected in new research.

Finally, our study has inadvertently highlighted the potential limitations of using the HRCS framework alone to categorise research. We advise researchers and funders using this framework to consider the implications for their own work and involvement of relevant stakeholders to supplement it.

## 5 Conclusion

There are many themes common to UK JLA research priorities that cut across multiple areas of health and care. To encourage new research, collaborations and research funding, we have published an interactive tool [22] to help researchers, funders and PLEx to explore these priority topics that transcend condition‐specific boundaries.

## Author Contributions

**Joanna C. Crocker:** conceptualization, investigation, writing–original draft, methodology, visualization, writing–review and editing, formal analysis, project administration, funding acquisition. **Lucy Moore:** formal analysis, writing–review and editing, investigation, methodology. **Margaret Ogden:** conceptualisation, writing–review and editing, methodology. **Sally Crowe:** conceptualisation, writing–review and editing, methodology, formal analysis. **Maaz Khan:** formal analysis, writing–review and editing, methodology. **Casper Schoemaker:** formal analysis, conceptualisation, investigation, writing–review and editing, methodology. **Noémi B. A. Roy:** conceptualisation, investigation, formal analysis, writing–review and editing, methodology. **Mark Taylor:** conceptualisation, investigation, methodology, writing–review and editing, formal analysis. **Toto Gronlund:** conceptualisation, investigation, writing–review and editing, formal analysis, methodology. **Teofila Bueser:** conceptualisation, investigation, writing–review and editing, formal analysis, methodology. **Madeline Tatum:** writing–review and editing, formal analysis. **Benjamin Davies:** writing–review and editing, conceptualisation, investigation, methodology. **Teresa Finlay:** conceptualisation, investigation, methodology, writing–review and editing.

## Conflicts of Interest

The authors declare no conflicts of interest.

## Supporting information

Supporting information.

Supporting information.

Supporting information.

## Data Availability

The data that support the findings of this study are available in overaching‐themes.zip and priority‐coding‐sheets.zip at https://www.phc.ox.ac.uk/research/resources/priorities-for-health-and-care-research/Priorities-for-Health-and-Care-Research. These data were derived from the following resources available in the public domain:  James Lind Alliance Top 10s of priorities for research, https://www.jla.nihr.ac.uk/top-10-priorities/.
