# Supplementary material for: Overarching Priorities for Health and Care Research in the United Kingdom: A Coproduced Synthesis of James Lind Alliance ‘Top 10s’
Source: Health Expect. 2024 Jun 19;27(3):e14096. doi: 10.1111/hex.14096 (PMC11187853; doi:10.1111/hex.14096)
Supplement: Supplementary file 1 — Supporting information. [file HEX-27-e14096-s001.docx]

## Guidance for coding JLA PSP priorities using the UK Health Research Classification System (HRCS)

We developed the following guidance for coders to use alongside the full HRCS [Health Category](https://hrcsonline.net/health-categories/) and [Research Activity](https://hrcsonline.net/research-activities/) frameworks, [introductory resources](https://hrcsonline.net/getting-started/) and [Guidance](https://hrcsonline.net/guidance/).

**General rules**

- Code clear themes/concepts only. If in doubt about the relevance of a secondary/tertiary code, either don’t use the code or flag it for discussion with your team.
- Try to code each concept within a priority only once, by choosing the ‘best fit’ (rather than assigning two different codes to the same concept). Use additional codes when the priority clearly contains two or more relevant concepts which have roughly equal importance.

**HRCS Health Category**

Definitions of each Health Category can be found here: <https://hrcsonline.net/health-categories/>

- Use one or two codes which best fit the PSP as a whole.
  - E.g. the Diabetes and Pregnancy PSP would be assigned two codes: “Metabolic and Endocrine” and “Reproductive health and childbirth”.
- Health Categories should **not** be assigned to reflect mechanisms of pathogenesis or the site of a disease. Choose the Health Category associated with the purpose of the investigation or the overarching main disease.
  - E.g. the Neuro-oncology PSP would be coded under “Cancer and neoplasms”, **not “**Neurology”.
- Use additional codes if there are other areas of health or disease referred to in the question / research priority.
  - E.g. In the Multiple Conditions in Later Life PSP, priority 8 “What are the most effective, cost effective and acceptable interventions to improve the psychological wellbeing of older people with multiple conditions?” would be coded under “Mental Health” (as well as “Generic Health Relevance” due to the PSP topic).
- For non-health related PSPs or priorities, select ‘Disputed Aetiology and Other’ in the appropriate field.

**HRCS Research Activity**

These codes should reflect the type of research activity required to address the research priority. There are 8 overarching codes and 48 subcodes, defined here: <https://hrcsonline.net/research-activities/> General information about assigning Research Activity can be found here: <https://hrcsonline.net/getting-started/general-approach-to-coding/assigning-research-activities/>

- Use the minimum number of codes to reflect the focus of the research priority. Use multiple codes only if you feel they should be given roughly equal weight/importance. For example:
  - “How can the best treatment for each individual patient with pancreatic cancer be identified (e.g. regarding surgery and chemotherapy)?” would be coded as 7.3 (Management & Decision Making) only (**not** 6.7 Surgery and 6.1 Pharmaceuticals).
  - “How can adverse effects and long-term damage from medicinal treatment be avoided?” is coded 7.1 (Individual care needs, which includes management of side-effects), **not** 6.1 Pharmaceuticals.
- Use full information on subcode webpage (including ‘Main inclusion criteria’ and ‘Advice on research activities’) to make coding decisions.
- If there is insufficient detail to assign a subcode, use the most relevant overarching code.
  - E.g. "What is the cause of pregnancy hypertension (including pre-eclampsia)?" would be coded under 2 (Aetiology).
- If the question fits **two** possible subcodes under one overarching code, use both subcodes. If the priority fits **more than two** possible subcodes under an overarching code, use the overarching code instead. E.g.:
  - “How can patients’ choices and shared decision making be enhanced?” would be 7.1 (Individual care needs) and 7.3 (Management and decision making).
  - “What is the natural history of DCM? What is the relationship between DCM and asymptomatic spinal cord compression or canal stenosis? What factors influence the natural history of the disease?" Could include subcodes 2.1, 2.2, 2.3 and/or 2.4, so would be coded as 2 (Aetiology).

**Advice on using specific Research Activity codes:**

- If there are non-health related priorities, or elements of priorities, which fall outside the scope of the HRCS Research Activity framework, code as ‘Other’.
  - E.g. for DCM PSP, “What strategies can be used to increase awareness and understanding of DCM amongst healthcare professionals and the general public?” would be coded as 7.3 (Management and decision making) **and** Other (to capture the general public element).
- Code 1 is for normal processes including ageing and pregnancy not linked to a condition or service use. E.g.:
  - “What are women’s experiences of labour and childbirth?” would be coded as 1.1.
  - “What are the experiences of women using obstetric services?” would be coded as 8.1 (which includes evaluation of service user experiences).
- Code 2 is for aetiology including development of disease.
  - E.g. “What is the natural history of degenerative cervical myelopathy?” would be coded as 2.
- Code 3 is for primary prevention only i.e. in healthy people or ‘at risk’ people without a pre-existing condition. It excludes secondary prevention research which will usually be under 6 (Treatment Evaluation) or 7 (Disease Management). E.g.:
  - “How can pregnancy hypertension (including pre-eclampsia) be prevented in a subsequent pregnancy?" would be coded under 3 (Prevention) because women with a history of hypertension are ‘at risk’ but no longer have an abnormal condition.
  - “How can we predict and prevent shorter term complications of pregnancy hypertension (including stillbirth, fetal growth restriction, neonatal death, progression to pre-eclampsia)?” would be coded under 7.1 and 7.3, because this question is about preventing complications of an existing condition.
- When ‘management strategy’ is referred to in a priority, this does not necessarily mean treatment, so consider coding under 7 (Management of Diseases and Conditions) rather than 5/6 (Development/Evaluation of Treatments and Therapeutic Interventions).
- When ‘treatment’ is referred to in a priority, this does not necessarily mean pharmaceutical treatment. Consider using overarching codes 5 or 6 (Development or Evaluation of Treatments and Therapeutic Interventions) rather than subcodes 5.1 or 6.1 (Development or Evaluation of Pharmaceuticals).
- Unspecified ‘topical’ products should generally be classed as pharmaceutical (5.1 or 6.1) and **not** complementary (5.8 or 6.8).
- Evaluation of nutritional supplements used as treatment for disease should be coded as 6.1 (Pharmaceuticals), **not** 3.3 (Nutrition and Chemoprevention).
  - E.g. “Does nutrition influence the survival and/or quality of life of patients with pancreatic cancer?” is coded as 6.1.
- When a priority is primarily focused on cost-effectiveness of a therapeutic intervention or management strategy, this should be coded under 8.2 (Health and welfare economics) as well as 6 (for therapeutic interventions) or 7 (for management strategies).
- Code 7.1 (individual care needs) includes studies assessing social care or health service needs. Code 8.1 (organisation and delivery of services) is about the provision and delivery of health and care services.
- Code 7.3 (management and decision making) includes studies about the decision making process in diagnosis and prognosis, as opposed to evaluating diagnostic or prognostic techniques (which would fall under code 4 - Detection and Diagnosis).
