# Supplementary material for: Overarching Priorities for Health and Care Research in the United Kingdom: A Coproduced Synthesis of James Lind Alliance ‘Top 10s’
Source: Health Expect. 2024 Jun 19;27(3):e14096. doi: 10.1111/hex.14096 (PMC11187853; doi:10.1111/hex.14096)
Supplement: Supplementary file 2 — Supporting information. [file HEX-27-e14096-s003.docx]

**Supplementary File 1: Topics excluded from analysis**

| **Topic** | **Reason for exclusion** |
| --- | --- |
| Research methods: quantitative vs. qualitative vs. mixed methods | Not relevant to main aim of project and difficult to assess. Many priorities could be addressed using multiple research methods. |
| Translational research / translation-ready priorities |  |
| Avoidable vs unavoidable health conditions | Too complex and subjective. |
| Individual risk factors (where individual can make a choice) versus societal/socio-economic risk factors (where individual does not have a choice; risk is determined by social situation) |  |
| Misalignment between original uncertainties (patient focused) and final priority (clinically focused) | Interesting but this is about the JLA process; not relevant to main aim of project. |
