# Supplementary material for: Overarching Priorities for Health and Care Research in the United Kingdom: A Coproduced Synthesis of James Lind Alliance ‘Top 10s’
Source: Health Expect. 2024 Jun 19;27(3):e14096. doi: 10.1111/hex.14096 (PMC11187853; doi:10.1111/hex.14096)
Supplement: Supplementary file 3 — Supporting information. [file HEX-27-e14096-s002.docx]

## Supplementary File 3: Stakeholder-generated codes and their application

| **Code name** | **Source topic*** | **Source stakeholder**** | **How applied to priorities** |
| --- | --- | --- | --- |
| Alcohol | Lifestyle factors | PLEx(AG), RF | Coded within HRCS Research Activity 1.2, 2.3, 3.1 |
| Caregivers & Families | Carer perspective / knowledge / views / engagement | PLEx(AG/W) | Keyword search: carer*, caregiver*, family, families, parent* |
| Communication & information sharing | Information sharing | PLEx(W) | Coded within HRCS Research Activity 3.1, 7.1, 7.3, 8.3 |
| Delay | Time and time points | PLEx(AG) | Keyword search: delay, wait*, time |
| Diet | Diet (tailored to specific condition) | PIS | Keyword search: diet, nutrition, food, eat, carbohydrate, fat, protein |
| Exercise | Exercise (tailored to specific condition) | PIS | Keyword search: exercise, physical activity, active |
| Fatigue | Fatigue | PLEx(AG) | Keyword search: fatigue |
| Health education | Health literacy and education | PLEx(W) | Coded within HRCS Research Activity 3.1, 7.1, 7.3 and Other |
| Health inequalities | Health inequity/inequalities | PLEx(W), RF | Keyword search: vulnerable, groups, under-served, unequal, inequit*, inequalit*. Search for protected characteristics: age, gender, marital/civil partnership status, disability, pregnancy/maternity, race, religion/belief, sex, sexual orientation. |
| Health literacy | Health literacy and education | PLEx(W) | Coded within HRCS Research Activity 3.1, 7.1 and Other |
| Inequality of access | Inequality of access to services | PLEx(W) | Coded within HRCS Research Activity 8.1 |
| Multi-morbidity | Multi-morbidity | JLA | Checked priorities coded under HRCS Generic Health Relevance or with 2+ Health Category codes |
| Pain | Pain | PLEx(AG/W) | Keyword search: pain |
| Pharmaceuticals | Medicines / pharmaceuticals | PLEx(W) | Combined HRCS Research Activity 5.1 & 6.1 |
| Physical environment | Physical environment | RF | Combined HRCS Research Activity 1.3, 2.2 & 3.2 |
| Place of care | Service delivery / place of delivery | PLEx(AG), RF | Coded within HRCS Research Activity 8.1 |
| Psychological impact of illness | Mental health/ psychological well-being | PLEx(W) | Coded within HRCS Research Activity 7.1 |
| Psychological risk factors | Mental health/ psychological well-being | PLEx(W) | Coded within HRCS Research Activity 1.2, 2.3, 3.1, 4.4 |
| Psychological, social, behavioural & economic determinants of health | Mental health/ psychological well-being; Social determinants of health / healthy life expectancy; Social science angle | PLEx(W) | Combined HRCS Research Activity 1.2, 2.3, 3.1, 4.3 |
| Public knowledge, views, attitudes & behaviour | Implications for wider public | AR, PLEx(AG) | Coded within HRCS Research Activity 2, 3 and Other |
| Quality of life | Quality of life | C | Coded within HRCS Research Activity 7.1 and COMET “Life Impact” domain |
| Research (design, methods, dissemination & implementation) | Evidence-based methods | AR | Combined HRCS Research Activity 8.3 & 8.4 |
| Resources and infrastructure | Pathway/process/systems engineering, complex systems | AR | Combined HRCS Research Activity 1.5, 2.6, 3.5, 4.5, 5.9, 6.9, 7.4, 8.5 |
| Self-management | Patient self-management | PLEx(AG) | Coded within HRCS Research Activity 7.1 |
| Sex & sexual health | Lifestyle factors | PLEx(AG), RF | Coded within HRCS Research Activity 1.2, 2.3, 3.1 |
| Shared decision-making | Shared decision-making | PLEx(W) | Coded within HRCS Research Activity 7.1 and 7.3 |
| Smoking | Lifestyle factors | PLEx(AG), RF | Coded within HRCS Research Activity 1.2, 2.3, 3.1 |
| Social | Social determinants of health / healthy life expectancy; Social science angle on health | PLEx(AG/W), RF | Coded within HRCS Research Activity 1.2, 2.3, 3.1, 4.4, 6.6, 7.1, 7.2, 8.1 |
| Substance misuse | Lifestyle factors | PLEx(AG), RF | Coded within HRCS Research Activity 1.2, 2.3, 3.1 |
| Surgery | Surgery / surgical interventions | PLEx(W) | Keyword search: surg*, operat*, transplant |
| Technology | Technology/digital health | AR | Keyword search: tech*, app*, media, digital, online, on-line, internet, tele*, remote, computer, phone |
| Transitions in care | Service delivery / place of delivery | PLEx(AG) | Coded within HRCS Research Activity 7 & 8 |
| Utilising patient/carer expertise | Experts by experience | PLEx(W) | Coded within HRCS Research Activity 7.1 & 7.3 |

***** Original topic suggested by a patient, carer, service user or other stakeholder

**AG = advisory group; AR = academic researcher; C = clinician; JLA = James Lind Alliance Coordinating Team; PIS = public involvement specialist; PLEx = Person with lived experience; RF = research funder; W = workshop
